# Associations between early marriage and preterm delivery: Evidence from lowland Nepal

**DOI:** 10.1002/ajhb.23709

**Published:** 2021-12-04

**Authors:** Faith A. Miller, Akanksha A. Marphatia, Jonathan C. Wells, Mario Cortina‐Borja, Dharma S. Manandhar, Naomi M. Saville

**Affiliations:** ^1^ Institute for Global Health (IGH) University College London (UCL) London UK; ^2^ Department of Geography University of Cambridge Cambridge UK; ^3^ Population, Policy and Practice Research and Teaching Department, Great Ormond Street Institute of Child Health (ICH) University College London (UCL) London UK; ^4^ Mother and Infant Research Activities (MIRA) Kathmandu Nepal

## Abstract

**Objectives:**

Preterm delivery (<37 weeks gestation) is the largest cause of child mortality worldwide. Marriage and pregnancy during adolescence have been associated with an increased risk of preterm delivery. We investigate independent associations of age at marriage and age at first pregnancy with preterm delivery in a cohort of women from rural lowland Nepal.

**Methods:**

We analyzed data from 17 974 women in the Low Birth Weight South Asia Trial. Logistic regression models tested associations of age at marriage and age at first pregnancy with preterm delivery, for primigravida (*n =* 6 243) and multigravida (*n =* 11 731) women. Models were adjusted for maternal education, maternal caste, and household asset score.

**Results:**

Ninety percent of participants had married at <18 years and 58% had their first pregnancy at <18 years. 20% of participants delivered preterm. Primigravida participants married at ≤14 years had higher odds of preterm delivery than those married ≥18 years, when adjusting for study design (adjusted odds ratio (aOR) 1.45, 95% CI: 1.15–1.83), confounders (aOR 1.28: 1.01–1.62) and confounders + age at pregnancy (aOR 1.29: 1.00–1.68). Associations were insignificant for multigravida women. No significant associations were observed between age at first pregnancy and preterm delivery.

**Discussion:**

In this population, early marriage, rather than pregnancy, is a risk factor for preterm delivery. We hypothesize that psychological stress, a driver of preterm delivery which is increased among those marrying young, rather than physiological immaturity, drives this association. Further research into the psychological consequences of child marriage in Nepal is needed.

## 1 INTRODUCTION

Preterm delivery refers to a delivery occurring before 37 weeks gestation (Althabe et al., 2012). Of the 15 million infants delivered preterm globally each year, an estimated 1 million die before the age of five years, making it the largest cause of child mortality worldwide (Chawanpaiboon et al., 2018; Lawn et al., 2005). Reducing preterm delivery rates is therefore a vital component of reducing infant and child mortality and morbidity, contributing towards the United Nations Sustainable Development Goal (SDG) 3, which aims to end all preventable deaths under the age of five years by 2030 (United Nations, 2020). From a physiological perspective, preterm delivery interrupts the continuous supply of nutrients via the placenta during a critical period of development (Czech‐Kowalska, 2020). Meeting nutritional demands through enteral feeding after birth is constrained by the immaturity of the gut in preterm infants, meaning they are prone to poor growth and development in early life (Zozaya et al., 2019). Furthermore, preterm infants have an increased risk of being exposed to inflammation, either prenatally or postnatally, which impairs neurodevelopment (Cappelletti et al., 2016; Lee et al., 2021). Prematurity therefore has long‐term consequences on the health and cognitive development of individuals into adulthood (Kajantie et al., 2010; MacKay et al., 2010; Markopoulou et al., 2019). This can cause families significant psychological and financial hardship (Moster et al., 2008; Rogers & Velten, 2011; Swamy et al., 2008).

Populations in Africa and Asia in particular bear the burden of preterm delivery, as these continents account for around 80% of cases globally (Chawanpaiboon et al., 2018). In South Asia, rates of children dying from complications following preterm delivery are declining at a slower rate compared to other causes of death regionally, meaning that the relative contribution of preterm delivery to childhood mortality has increased over the past 20 years (Liu et al., 2016). Recent efforts have improved the survival of premature infants, however in the Global South, particularly in remote and rural regions, limited access to medical facilities or skilled birth attendance means these improvements are not universal (Choulagai et al., 2017; Iams et al., 2008). Therefore, understanding the risk factors for and mechanisms involved in preterm delivery in these regions are key for prevention and related care.

There are several biological and social factors which have been associated with preterm delivery, such as a young age at pregnancy (Althabe et al., 2015; Ganchimeg et al., 2014; Gurung et al., 2020; Kumar et al., 2018; Stewart et al., 2007). This is very relevant in South Asia where an estimated 30% of girls give birth before their 18th birthday (Scott et al., 2020). In South Asia, marriage is the main context for sexual intercourse and therefore a young age at marriage is a key determinant of the age of first childbirth (Ministry of Health and Population (MOHP), 2017). Child marriage describes a marriage in which one or both spouses are below the age of 18 years, and is a fundamental violation of human rights (UN General Assembly, 1948; United Nations Population Fund, 2012). While the practice has decreased globally over recent decades, it remains prevalent in South Asia, where over half of marriages still take place during childhood, however the prevalence of child marriage varies greatly between regions (Scott et al., 2020; UNICEF, 2018, 2017). Given the scale of the practice of child marriage, understanding the associated consequences is a priority.

Marriage is associated with a range of lifestyle changes and new responsibilities, which present emotional, social and financial challenges, particularly during adolescence (Marphatia et al., 2017; Nour, 2009). The emotional consequences of child marriage are especially severe for girls (Raj et al., 2018). In South Asia, marriage generally requires girls to move in with their new husband's family and take on a new position of deference in the household (Harris‐Fry et al., 2018; Marphatia et al., 2017). Marriage also typically marks an end to a girl's formal education, limiting their personal freedom (Field & Ambrus, 2008; Marphatia et al., 2020; Sekine & Hodgkin, 2017). In this setting, girls with a lower level of education have reduced access to antenatal services and are less likely to participate in household decision making, including decisions regarding their own health (Ministry of Health and Population (MOHP), 2012; Sekine & Carter, 2019). Furthermore, those marrying during childhood have an increased risk of experiencing intimate partner violence (IPV; Kidman, 2017). Young brides in South Asia also report lower use of contraception and higher rates of pregnancy termination than those marrying in adulthood, highlighting the adverse reproductive health sequelae (Godha et al., 2013; Raj & Boehmere, 2013). These consequences are not short lived; emotional distress, lack of schooling, reduced access to healthcare, IPV, and poor reproductive health have all been associated with preterm delivery, meaning children born to mothers married during childhood are likely to be more vulnerable to the associated adverse health effects (Baer et al., 2019; Dunkel Schetter, 2011; Efevbera et al., 2017; Franck et al., 2020; Walani, 2020). Despite this, there is a dearth of empirical evidence on maternal health outcomes following child marriage (Godha et al., 2013).

Previous studies have focused on the consequences of child marriage within the context of early childbearing, viewing child marriage as a gateway to early pregnancy (Godha et al., 2013; Mathur et al., 2003; Nasrullah et al., 2014; Paul, 2020; Rahman et al., 2018). While adverse consequences of early pregnancy have been identified, there is considerable debate around whether these are the result of the mothers' biological immaturity, or stresses associated with socio‐economic factors or dynamics of the marital household (Gurung et al., 2020; Jiang et al., 2018; Maharjan et al., 2019; Shrestha et al., 2010; Stewart et al., 2007). Most likely, a combination of the two issues is relevant, particularly in cases of child marriage. In South Asia, pregnancy is almost always preceded by marriage and therefore these two exposures are closely intertwined. However, they remain separate events with individual drivers and consequences (Adhikari & Bott, 2003; Marphatia, Wells, et al., 2021). The interval between a girl's age at marriage and first pregnancy differs according to their age at marriage, as well as other factors which may delay the consummation of the marriage or influence their use of contraceptives, such as where they live, their ethnicity, educational status or religion (Gubhaju, 2009; Staveteig et al., 2018). Furthermore, as discussed above, a girl's age at marriage is associated with their access to healthcare, level of education and social engagement, which may impact their reproductive and maternal health through mechanisms independent of their age at pregnancy. Elucidating the consequences of both early marriage and early pregnancy is key to determine where best to focus efforts to improve maternal and child health. Furthermore, previous studies in this field have been limited by small sample sizes and low rates of child marriage (Huang et al., 2021; Pandya & Bhanderi, 2015; Rahman et al., 2018). This study uses data from the Low Birth Weight South Asia Trial (LBWSAT) to investigate the independent associations of age at marriage and first pregnancy with preterm delivery in rural lowland Nepal. Almost 90% of the LBWSAT participants were married during childhood (<18 years), making this dataset particularly well suited to studying its consequences (Marphatia, Saville, Manandhar, Amable, et al., 2021; Marphatia, Saville, Manandhar, Cortina‐Borja, et al., 2021). Analysis of primigravida and multigravida women separately examines whether any observed associations are restricted to the first pregnancy, or if longer term sequelae persist in subsequent pregnancies. This analysis also provides an insight into the mechanisms involved in preterm delivery.

## 2 METHODS

### 2.1 Study context

This analysis used data from the LBWSAT, a non‐blinded, cluster randomized controlled trial conducted in the Dhanusha and Mahottari districts of Nepal. The LBWSAT assessed the impact of community‐based participatory learning and action (PLA) women's groups, with and without food or cash transfers, on birth weight and infant weight‐for‐age *z* (WAZ)‐scores. All married women and girls aged 10–49 years across 80 village clusters were invited to take part in the trial, permitted they or their husband had not undergone surgical family planning (Saville et al., 2018). About 63 308 participants consented to menstrual monitoring from 80 randomized village development committee (VDC) clusters, and 25 090 pregnancies were detected between December 2013 and February 2015 (Saville et al., 2018). VDC clusters were randomly assigned to one of four interventions: behavioral change PLA groups, PLA groups + cash given to pregnant participants, PLA groups + “Super Cereal” supplement given to pregnant participants, or existing government programmes. The detailed study protocol and primary outcome results have been published previously (Saville et al., 2016, 2018; Style et al., 2017).

Informed written consent was taken from all participants in this study, and their guardians if they were aged <18 years. Research ethics approval was obtained from the Nepal Health Research Council (NHRC) (108/2012) and the University College London (UCL) Ethical Review Committee (4198/001) for primary data collection, and from the NHRC (292/2018) and the UCL Ethical Review Committee (0326/015) for the secondary analyses presented in this article.

### 2.2 Variable selection

The outcome variable for this analysis was the occurrence of preterm delivery (<37 weeks/259 days gestation). Gestation length (GL) was calculated as the time between last menstrual period (LMP) and date of delivery. LMP was determined using maternal recall for 18 306 pregnancies and using ultrasound for 1321 pregnancies where available. Agreement analysis between GL determined by ultrasound and maternal recall was undertaken by calculating Pearson's correlation coefficient and Cronbach's alpha for GL categories. The binary preterm delivery variable was selected rather than a continuous GL variable to align with other research and to minimize the influence of inaccuracies in maternal recall of LMP.

The primary exposure variables for this analysis were the participant's age at marriage and age at first pregnancy. In this trial's setting, most people count their age in running years rather than completed years. Therefore, age at marriage and first pregnancy were collected as integer values in running years and converted to completed years (running years −1) for analysis. To investigate the importance of policy aiming to reduce childhood marriage and motherhood, and based on the distribution and pattern of data in our sample, age at marriage was coded into four groups: ≤14 years, 15 years, 16–17 years, and ≥18 years, while age at first pregnancy was coded into three groups: ≤15 years, 16–17 years, and ≥18 years.

Using a priori knowledge, a directed acyclic graph (DAG) was constructed using DAGitty to identify the minimal sufficient adjustment variables to account for confounding (Textor et al., 2011; Supplemental Figures 1–4). The arrows in the DAG were entered to represent the hypothesized direct causal effect of one variable on another. Variables that are hypothesized to be directly antecedent to the exposure (age at marriage or age at pregnancy) and outcome (preterm birth) are indicated in pink and identified as a confounder (Hernán et al., 2002). This approach identified maternal education, maternal caste group and material household assets as confounders for the association between age at marriage and preterm delivery, as these factors are hypothesized to have a causal effect on both exposure and outcome (Supplementary Figures 1 and 2). For the association between age at first pregnancy and preterm delivery, the same factors plus age at marriage were identified as confounders (Supplementary Figures 3 and 4).

Maternal education was coded into four levels based on the Nepali education system and the distribution of data in our sample: no formal education, primary (1–5 years), lower secondary (6–8 years) and secondary or higher (≥9 years). The household asset score was determined using principal component analysis, reflecting ownership of consumer goods such as a color television, motorbike, or computer, land ownership and household infrastructure (Saville et al., 2018). Maternal caste was grouped into three groups: disadvantaged comprising of Dalit and Muslim, middle comprising of Janajati and other Terai castes, and advantaged comprising of Yadav and Brahmin.

Maternal weight and mid‐upper arm circumference (MUAC) were measured in early pregnancy (8–30 weeks). Maternal height was measured in early pregnancy, and endpoint where earlier height was missing. Body mass index (BMI) was subsequently calculated using early pregnancy weight and height collected at either timepoint, and adjusted for gestational age. However, these measurements were only available for a sub‐set of women. Season of birth and sex of the baby was determined at birth (within 42 days). Maternal age at current pregnancy was also collected at baseline for additional analysis in multigravida women, categorized into six categories: <18 years, 18–20 years, 21–23 years, 24–26 years, 27–29 years, and ≥30 years.

### 2.3 Statistical methods

The characteristics of primigravida and multigravida participants were described and compared using chi‐squared tests. The characteristics of participants with missing and complete GL data were also compared using chi‐squared tests. The relationship between age at first pregnancy and age at marriage was explored visually by generating heat tables in Microsoft Excel (Microsoft Corporation, 2018) and a correspondence analysis plot (“mcaplot” command in Stata). Correspondence analysis represents contingency tables as a low‐dimension geometric map of the association, representing the closeness between rows (age at first pregnancy) and columns (age at marriage; Greenacre, 1992).

Mixed‐effects logistic regression models were fitted to assess associations of the exposures with preterm delivery, with the VDC cluster included as a random effect term on the intercept (“xtlogit” function in Stata). Minimally adjusted models adjusted for study design, including the random effect for cluster and fixed effects for study arm and strata. Fully adjusted models adjusted for study design plus additional confounders identified using the DAG. Missingness patterns of variables were identified and multivariate imputation using chained equations (MICE) was applied to deal with missing data (“mi” function in Stata; 30 completed datasets; van Buuren & Groothuis‐Oudshoorn, 2011). To account for the multi‐level nature of the data, the imputation was executed stratifying for each study arm. Models were also run on un‐imputed data as a sensitivity analysis. All models analyzed the association between exposures and preterm delivery for primigravida participants and multigravida participants separately.

To determine the potential for mediation by age at pregnancy in the association between age at marriage and preterm delivery, additional models were constructed. These included age at current pregnancy as a covariate in the model for the association between age at marriage and preterm delivery, and removed age at marriage as a covariate in the model for the association between age at first pregnancy and preterm delivery. Further models were also constructed on the subsample of women with data on maternal height, BMI and MUAC to investigate the role of markers of maternal nutritional status. As rates of child marriage are gradually declining over time, additional models including only participants marrying within the last 5 years were constructed to rule out the potential bias of mothers marrying young being older.

All models report adjusted odds ratios (aOR) and 95% confidence intervals (CI). Statistical analyses were performed in Stata IC 16.1 (StataCorp, 2019).

## 3 DATA CLEANING

**FIGURE 1:**
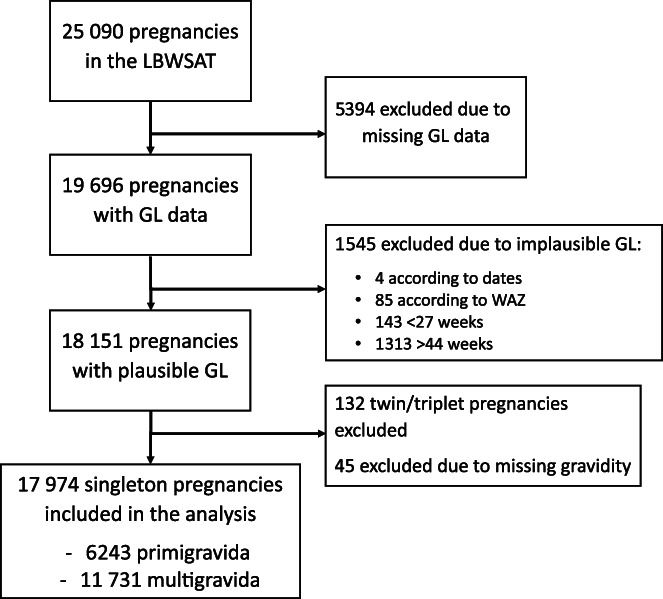
Participant flow diagram showing exclusions undertaken during data cleaning. Data cleaning protocol for this analysis. Briefly, exclusions were made due to erroneous dates, babies having gestational age‐adjusted weight‐for‐age z‐scores outside of the World Health Organization z‐score cut‐offs, erroneous gestation length (<27 weeks/>44 weeks), and multiple pregnancies. Abbreviations: LBWSAT, low birth weight South Asia Trial; GL, gestation length; WAZ; weight‐for‐age z‐scores

Of the 63 308 participants recruited for menstrual monitoring, there were 25 090 pregnancies during the trial period (Saville et al., 2018). Figure 1 demonstrates the cleaning of GL data for this analysis. About 5 394 participants (21%) with missing GL data were excluded. Participants with a GL out by ± ≥365 days (*n =* 60) were flagged as a data entry error and corrected accordingly, with four excluded due to erroneous dates.

Standardized WAZ‐scores were calculated using the “zanthro” Stata package which adjusts for gestational age (Vidmar et al., 2013). Analysis of *z*‐score distribution according to gestational age identified a bimodal birthweight distribution for preterm infants, as reported in previous studies (Haglund, 2007; Parker & Schoendorf, 2002). Erroneous GLs were identified as outliers using the World Health Organization (WHO) recommended *z*‐score cut‐off of +5 and their GL recoded to term (280 days). Weight‐for‐age *z*‐scores were then re‐calculated and accepted if they were now within range (−6/+5). All participants with a *z*‐score outside of this range were excluded. Additionally, preterm live births with a WAZ‐score above +3 who were enrolled >120 days after LMP were excluded due to suspected errors in maternal recall of LMP. Eighty‐five pregnancies were excluded due to erroneous WAZ‐scores (<1%).

All live births with a GL outside of the 27–44 week range were also excluded as GL was implausible (1456 participants: 6%). Forty‐five participants were excluded for having missing data on gravidity. One hundred and thirty‐two twin/triplet pregnancies were also excluded. Following data cleaning, 17 974 participants were included in the analysis. Supplemental Table 1 compares participants with complete GL data to those with missing and excluded GL data. Significant differences (chi‐squared tests; *p <* .05) between those with and without missing data were observed according to age at pregnancy, age at marriage, caste group, education level, study arm, and household asset score. Significant differences (chi‐squared tests; *p <* .05) in caste group, education level, study arm, season of birth, sex of the infant, and household asset score were observed between those with and without excluded data.

## 4 RESULTS

We included 6243 primigravida and 11 731 multigravida participants in this analysis. Table 1 displays and compares the characteristics of primigravida and multigravida participants separately. Twenty percent of primigravida and multigravida participants delivered preterm. 49% of primigravida participants and 63% of multigravida participants had their first pregnancy at <18 years (*p <* .001), while 86% of primigravida participants and 92% of multigravida participants had married at <18 years (*p <* .001).

**TABLE 1:** Study characteristics for primigravida and multigravida participants

	Primigravida participants included in analysis (%)	Multigravida participants included in analysis (%)	*p‐*value for difference
	*n =* 6243	*n =* 11 731	
Gestational age at delivery			
Term delivery	80.0	80.4	
Preterm delivery	20.0	19.6	.52
Missing	0.0	0.0	
Maternal age at first pregnancy			
≤15 y	8.4	25.6	
16–17 y	40.8	37.7	
≥18 y	50.8	36.7	<.001
Missing	0.0	3.1	
Maternal age at marriage			
≤14 y	20.3	41.5	
15 y	24.2	27.1	
16–17 y	41.2	23.3	
≥18 y	14.3	8.0	<.001
Missing	13.8	20.8	
Caste three groups			
Dalit/Muslim – Disadvantaged	32.0	36.3	
Janajati/Other Terai castes – Middle	44.3	42.4	
Yadav/Brahmin – Advantaged	23.7	21.3	<.001
Missing	0.0	0.0	
Household asset score			
1 – Most deprived	15.8	22.1	
2	18.6	21.2	
3	20.4	20.2	
4	22.1	19.0	
5 – Least deprived	23.1	17.6	<.001
Missing	1.4	1.0	
Mother education level			
Never went to school	49.3	72.9	
Primary	11.7	9.5	
Lower secondary	15.3	7.1	
Secondary and above	23.6	10.6	<.001
Missing	0.2	0.0	
Study arm woman enrolled in			
Control	23.1	22.2	
PLA group	23.3	23.7	
Cash	27.8	27.6	
Food	25.8	26.5	.43
Missing	0.0	0.0	
Season of birth			
Winter: Mid‐December to mid‐March	31.8	27.7	
Spring: Mid‐March to mid‐June	20.3	22.7	
Monsoon: Mid‐June to mid‐September	25.5	27.4	
Autumn: Mid‐September to mid‐November	22.4%	22.2	<.001
Missing	1.8	2.2	
Sex of infant			
Boy	52.0	53.7	
Girl	48.0	46.3	.037
Missing	1.1	0.9	

*Note*: Characteristics of primigravida and multigravida participants, compared using Pearson's chi‐squared tests.

Abbreviations: PLA, participatory learning and action groups; y, years of age.

[Correction to Table 1 added on 30 December 2021, after first online publication. The values in the rows titled Missing have been right‐aligned.]

**FIGURE 2:**
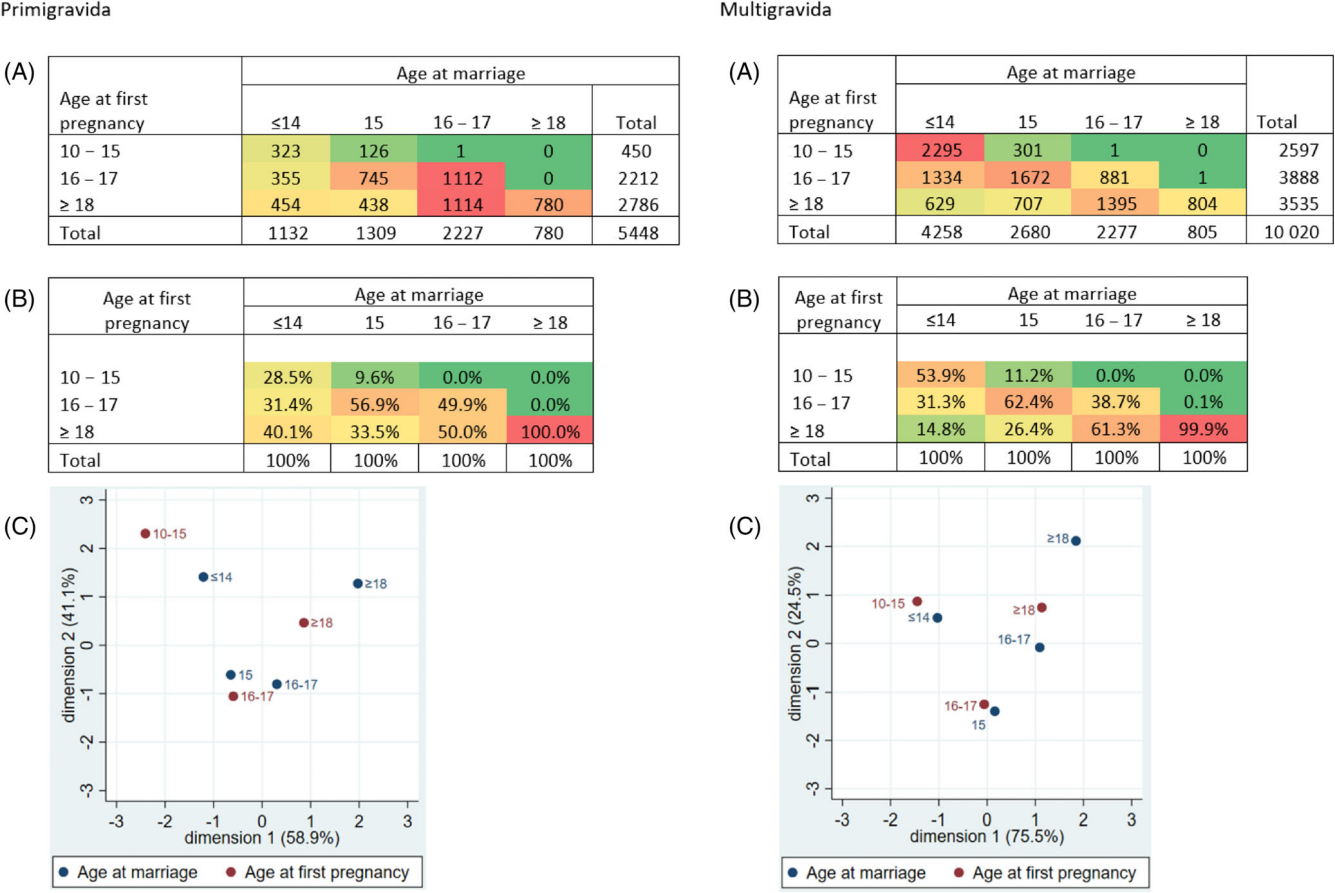
Heat tables for the association between age at marriage and age at first pregnancy. Heat tables for the association between age at marriage and age at first pregnancy for primigravida participants and multigravida participants in (A) absolute numbers, and (B) as a percentage within age at marriage. Correspondence analysis is represented in the bio plots (C). The % on the *x* and *y* axis represent the % of variance explained in a principal components analysis. Abbreviation: y, years of age

Figure 2 illustrates the association between age at marriage and age at first pregnancy in our study population. In the heat tables, green shading indicates the lowest level of association, and red shading indicates the highest. 54% of multigravida participants marrying at ages ≤14 years had their first pregnancy by the age of 15 years, 31% at ages 16–17 years, and 15% aged 18 years or older. 29% of primigravida participants marrying at ages ≤14 years had their first pregnancy by the age of 15 years, 31% at ages 16–17 years, and 40% aged 18 years or older. The correspondence analysis bio plots represent the closeness between age at marriage and age at first pregnancy. The axes demonstrate the proportion of variance explained in a principal components analysis; for primigravida participants, the first and second axes concentrate 59% and 41% of variability, for multigravida they concentrate 76% and 24%. As both variables are ranked by age along the x axes, the first correspondence component reflects increasing age. The second correspondence component has no clear interpretation, reflecting variability in the timing between marriage and first pregnancy.

**FIGURE 3:**
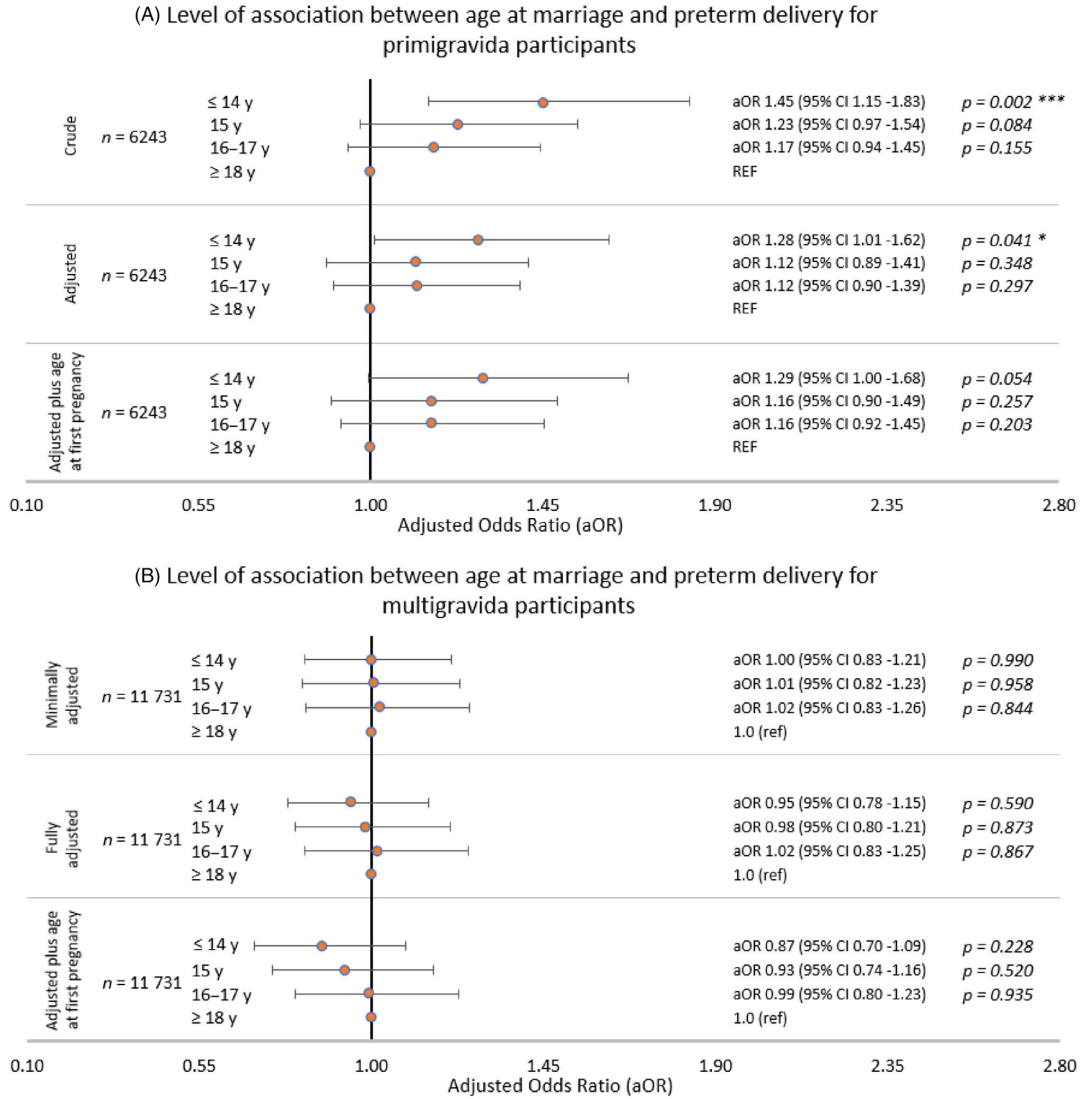
Adjusted odds ratios (aOR) for the association of age at marriage with preterm delivery. Association between preterm delivery and age at marriage for (A) primigravida participants and (B) multigravida participants. Core confounders identified using a directed acyclic graph were maternal caste, maternal education, and household asset score. Minimally adjusted: Adjusted only for random effect of cluster and fixed effect of study arm and strata, using multivariate imputation by chained equations. Fully adjusted: Adjusted for cluster, study arm, strata and core confounders, using multivariate imputation by chained equations. Fully adjusted plus age at pregnancy: Adjusted for cluster, study arm and strata and core confounders + age at pregnancy, using multivariate imputation by chained equations. Abbreviations: aOR, adjusted odds ratio; y, years of age. *p*‐value significance: * <.05, ***<.01

Table 2 presents preterm birth rates per 1000 deliveries by categories of age at marriage and age at first pregnancy, showing higher unadjusted rates of preterm delivery among primigravida participants marrying young compared to multigravida. Figure 3 displays the aOR for the association between age at marriage and preterm delivery. Primigravida participants married at ages ≤14 years had significantly higher odds of delivering preterm than those married aged ≥18 years in the minimally adjusted models (Figure 2; aOR 1.45, 95% CI: 1.15–1.83, *p =* .002). This significance was maintained when adjusting for confounders (aOR 1.28, 95% CI: 1.01–1.62, *p =* .041). Inclusion of age at pregnancy as a potential mediator slightly reduced the significance of the association between marrying ≤14 years of age and preterm delivery, but did not change the effect size (aOR 1.29, 95% CI: 1.00–1.68, *p =* .054). While there were trends towards a higher odds of preterm delivery for primigravida participants married aged 15 or 16–17 years, these associations were insignificant for all models. There were no significant associations observed between age at marriage and preterm delivery when analyzing multigravida participants. Results were not changed by the use of multivariate imputation, as shown by the estimates from nonimputed models in Supplemental Table 2.

**TABLE 2:** Rates of preterm delivery according to each category of age at marriage and age at first pregnancy

	Rate of preterm delivery/1000 deliveries	
	Primigravida	Multigravida
Age at marriage		
≤14 y	220	186
15 y	197	193
16–17 y	191	196
≥18 y	165	190
Age at first pregnancy		
≤15 y	226	199
16–17 y	193	196
≥18 y	202	190

*Note*: Rates of preterm delivery per 1000 live deliveries for each category for age at marriage and age at first pregnancy.

Abbreviation: y, years of age.

**FIGURE 4:**
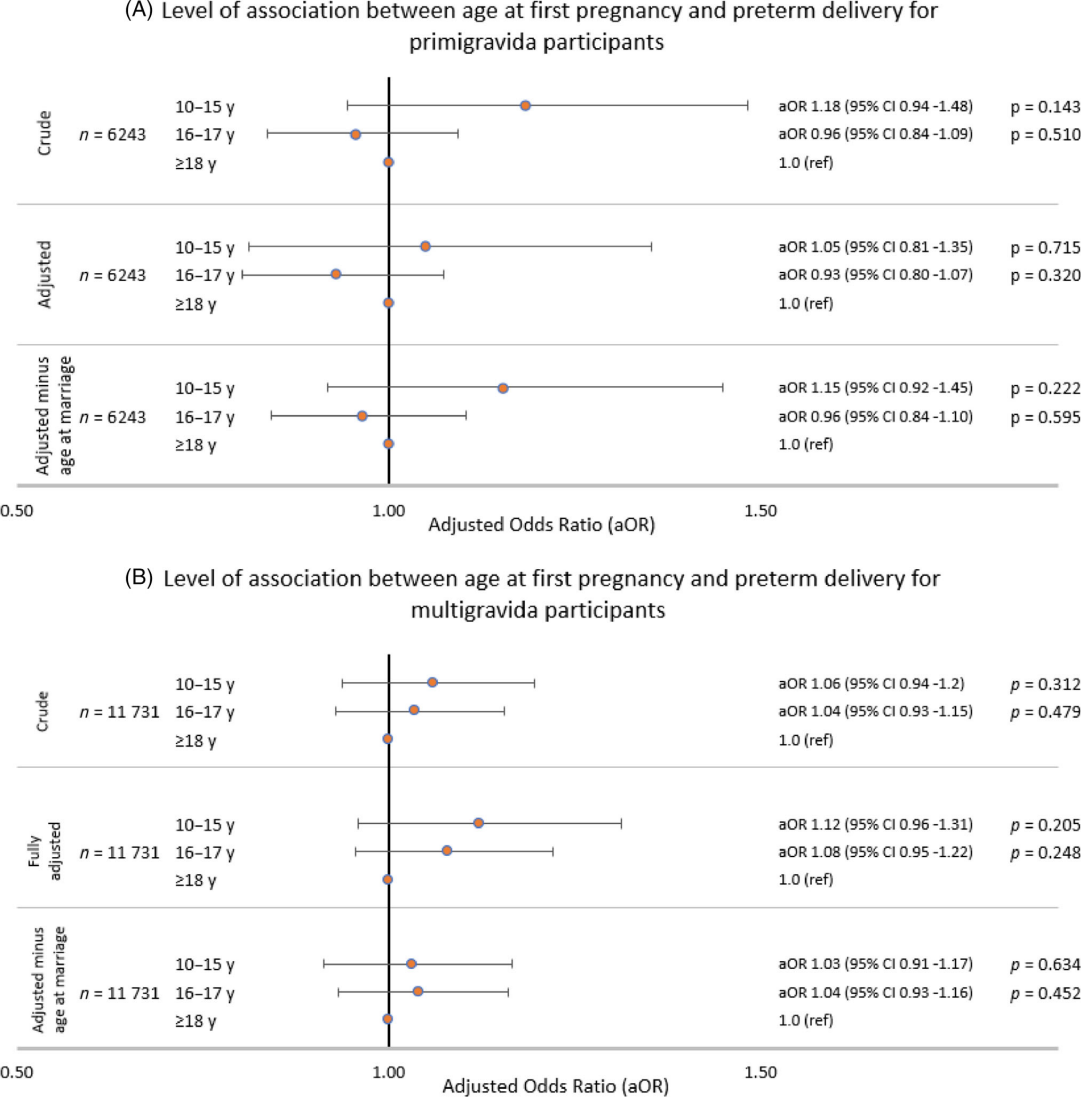
Adjusted odds ratios (aOR) for the association of age at first pregnancy with preterm delivery. Association between preterm delivery and age at first pregnancy for (A) primigravida participants and (B) multigravida participants. Core confounders identified using a directed acyclic graph were maternal caste, maternal education, household asset score and age at marriage. Minimally adjusted: Adjusted only for random effect of cluster and fixed effect of study arm and strata, using multiple imputation by chained equations. Fully adjusted: Adjusted for cluster, study arm, strata and confounders, using multivariate imputation by chained equations. Fully adjusted minus age at marriage: Adjusted for cluster, study arm, strata and confounders (minus age at marriage), using multivariate imputation by chained equations. Abbreviations: aOR, adjusted odds ratio; y, years of age. *p*‐value significance: *<.05, ***<.01

Figure 4 displays the aOR for the association between age at first pregnancy and preterm delivery. Primigravida participants giving birth aged ≤15 years had a nonsignificant higher odds of preterm delivery in the minimally adjusted model (aOR 1.18, 95% CI: 0.94, 1.48), however this association was substantially attenuated in fully adjusted models. No significant associations were observed between age at first pregnancy and preterm delivery in multigravida participants in any model (Figure 4).

Supplemental Table 3 displays investigations into whether markers of nutritional status explain our observed associations. While models including BMI and MUAC were limited by a high amount of missing data, a significant increase in the odds of delivering preterm for primigravida participants married <14 years was maintained when adjusting for BMI (*n* = 1074; aOR 2.15, 95% CI: 1.13–4.09), MUAC (*n* = 1074; aOR 2.06, 95% CI: 1.08–3.94) and height (*n* = 3795; aOR 1.36, 95% CI: 1.01–1.82), in addition to core confounders (maternal education, maternal caste group and material household assets). Supplemental Table 4 displays the same models, excluding markers of nutritional status, for the same cohort to compare.

Supplemental Table 5 displays models run on the cohort of participants married ≤5 years ago, and rules out the potential bias of mothers marrying young being older (due to a reduction in child marriage over time).

## 5 DISCUSSION

We found that the odds of delivering preterm are 1.3 times higher in primigravida participants marrying at ages ≤14 years compared to those marrying during adulthood. This association was not sustained in subsequent pregnancies for multigravida participants. Furthermore, our study found no significant association between age at first pregnancy and preterm delivery, even for primigravida participants. This suggests the association we observed between early marriage and preterm delivery is driven by mechanisms outside of the physiological consequences of early pregnancy in this population. Indeed, 40% of primigravida participants married at ages ≤14 years had their first pregnancy in adulthood. Few studies have looked directly at the association between child marriage and preterm delivery, and those doing so have found a stronger association than ours (between two and four times higher odds in adjusted and unadjusted models, respectively), but were limited by their small sample size (Huang et al., 2021; Pandya & Bhanderi, 2015; Rahman et al., 2018).

Our results suggest that efforts to delay marriage until adulthood among those most vulnerable to early child marriage has the potential to reduce their odds of preterm delivery by a quarter and improve rates of childhood survival. This is before considering the additional benefits of delaying marriage for the mother, such as improved education and mental health, which are closely intertwined with child marriage (Marphatia, Saville, Manandhar, Amable, et al., 2021). Moreover, reducing the risk of preterm birth by delaying marriage could also benefit the long‐term health of the offspring and improve the accumulation of human capital and educational attainment (MacKay et al., 2010; Marphatia, Reid, et al., 2019). Since education in the next generation remains critical for preventing child marriage, these associations suggest reducing early marriage could help disrupt inter‐generational cycles of disadvantage (Marphatia, Reid, et al., 2019; Marphatia, Saville, et al., 2019; Wells et al., 2019).

### 5.1 Psychological stress

We propose several potential explanations for the association observed between a young age at marriage and preterm delivery. First, while marriage is considered a successful outcome in Nepal, the transition to married life is often a time of psychological stress. Young brides move from their parental to their marital home, where they must adapt and conform to the expectations and requests of their new husband's family (Davis, 2009; Gram, 2018). As a result, they are at risk of becoming overworked, under‐nourished, and vulnerable to physical and emotional abuse (Gram, 2018). This transition is distressing, particularly during early adolescence when their young age contributes to their sense of disempowerment (Jensen & Thornton, 2003; Parsons et al., 2015). Also, younger girls have a stronger emotional attachment to their own families, which may make the transition more difficult and impact on relations with their in‐laws (Moretti & Peled, 2004; Palriwala, 1993). While large studies on the emotional consequences of child marriage are lacking, there is increasing evidence that adolescents marrying young experience negative mental health consequences (Burgess et al., 2021; Kidman, 2017; Kumar et al., 2018; Oshiro et al., 2011; Raj et al., 2018; Sezgin & Punamäki, 2020). However, the differences in social and cultural contexts between studies limits comparison, and research on the mental health consequences of child marriage in Nepal is limited (Chaulagain et al., 2019). Furthermore, adolescence is a time of critical development with regards to one's mental health, likely amplified while adapting to the practical, cultural and religious expectations of marriage during adolescence (Powers et al., 1989; Rose‐Clarke et al., 2021). Gender norms which drive the practice of child marriage may also play a role in normalizing physical and emotional abuse (Le Strat et al., 2011; Nour, 2009). Girls marrying young are at a higher risk of experiencing IPV and, due to their reliance on their husband's family, are less able to speak out against it (Kanesathasan et al., 2008). Emotional stress is a well‐known risk factor for preterm delivery, mediated by neuroendocrine and inflammatory mechanisms and changes in health behaviors (Dunkel Schetter, 2011; Gennaro & Hennessy, 2003), therefore it represents a plausible explanation for the association we observed between child marriage and preterm delivery. Future research into the negative reproductive health sequelae of child marriage would benefit from research into the consequences associated with mental health and stress.

### 5.2 Undernutrition

Young brides are also commonly undernourished (Goli et al., 2015). Markers of undernutrition were included in our DAG and did not emerge as key confounders (Supplemental Figures 1–4), however the DAG is a simplification of reality and undernutrition remains a plausible mechanism for the association between child marriage and preterm delivery. While undernutrition is prevalent within Nepali children generally, the intrahousehold allocation of food discriminates against pregnant women and girls in their marital home in particular (Chalise et al., 2018; Harris‐Fry et al., 2018; Palriwala, 1993). In addition, their mental health may impact on their appetite (Chorghade et al., 2006). A shorter stature, a marker of undernutrition in childhood, has indeed been associated with a young age at marriage and first pregnancy (Marphatia, Saville, Manandhar, Amable, et al., 2021; Marphatia, Saville, Manandhar, Cortina‐Borja, et al., 2021). Nutritional status has been associated with adverse pregnancy outcomes, including preterm delivery (Bloomfield, 2011; Kramer et al., 1992; Nsereko et al., 2020; Shah et al., 2014). This may be compounded by the increased risk of undernourishment observed in adolescent pregnancies, as mother and fetus compete for nutrients, undermining the growth of each (Goli et al., 2015; Marphatia, Saville, Manandhar, Cortina‐Borja, et al., 2021). Furthermore, seasonal patterns in preterm deliveries exist within this population, which may be mediated by seasonal variability in food availability and nutrition (Hughes et al., 2014; Saville et al., 2021). We undertook some analysis adjusting models for nutritional status (height, BMI and MUAC; Supplemental Tables 3 and 4), finding that a significant association remained between child marriage and preterm delivery. However, data on nutritional status was only available for a small subset of our sample (between 17% and 78% of our study population), limiting our conclusions from this analysis. The potential contribution of nutritional status to the association between early marriage and preterm delivery merits further research.

### 5.3 Education

Our models adjusted for a girl's level of formal education, however there is a wider education which occurs during adolescence as a part of their transition to womanhood, which is not accounted for in formal education. Girls develop a sense of their identity and place in society during adolescence, undergoing vast emotional and psychosocial development (Marphatia et al., 2017). They also gain an improved understanding of their reproductive and sexual health through interactions with their peer groups (Mathur et al., 2003). As a girl's husband and in‐laws generally determine their participation in society, childhood marriage tends to isolate girls from their peers and wider society, reducing their social capital and networks (Parsons et al., 2015). Therefore, girls marrying at younger ages miss out on an informal education from their peers, which could have consequences for their reproductive health (Nour, 2009). This may contribute to the reduced knowledge of sexual health and uptake of contraception in girls marrying young and the increased risk of developing an sexually transmitted infection (STI; Nour, 2006; Raj et al., 2018; Raj & Boehmere, 2013). While the risk of contracting an STI is lower within marriage, an unmet need for contraception remains, particularly within couples where one is a migrant worker, among whom sexual risk taking is more common (Mehata et al., 2020; Ministry of Health and Population (MOHP), 2017; Simkhada et al., 2017). As STIs are associated with an increased risk of preterm delivery, this may contribute to the association observed within the LBWSAT cohort (Johnson et al., 2011; Mann et al., 2010; Moodley & Sturm, 2000).

### 5.4 Compounding vulnerabilities

Socioeconomic vulnerabilities are often cited as a possible cause of the association between child marriage and adverse maternal health outcomes (Ganchimeg et al., 2014; United Nations Population Fund, 2012). Indeed, analysis of the LBWSAT cohort has found that a woman's age at marriage determines their access to different forms of capital (Marphatia, Saville, Manandhar, Amable, et al., 2021). However, our models adjusted for caste group, material household assets and maternal education level, and still found a significant association for marrying young outside of these socioeconomic factors. More broadly, the social context for child marriage in Nepal is complex, with different studies finding contrasting associations between socioeconomic status and child marriage (Aryal, 2007; Pandey, 2017; Raj et al., 2014). However, as maternal health care utilization is lower among those married during childhood, it is likely that compounding social vulnerabilities negatively impact health care access following child marriage (Sekine & Carter, 2019).

### 5.5 Age at pregnancy

Previous research has focused on the negative reproductive health consequences of child marriage within the context of early childbearing (Gurung et al., 2020; Rahman et al., 2018). While the significance of our observed association between age at marriage and preterm delivery is reduced when adjusting for age at pregnancy (*p =* .054), the change in significance is small and the effect size remains constant. This suggests that the effect of age at marriage is not mediated by age at pregnancy, and that an independent effect of age at marriage exists. The two exposures are closely intwined; child brides are less likely to use contraception prior to their first pregnancy, often forcing them into early motherhood (Godha et al., 2013; Raj, 2010). In these circumstances, there are a range of consequences of girls entering into pregnancy while they are physiologically immature themselves, due to the nutritional competition between mother and fetus (Gibbs et al., 2012; King, 2003). However, 40% of our primigravida participants marrying at age ≤14 years gave birth aged 18 years or older. In the bio plots in Figure 2 we observe a closer relationship between marriage at ages ≤14 years and first pregnancy at 10–15 years for multigravida participants, whereas this age at marriage group among primigravida participants is more evenly distributed between all three categories of age at pregnancy. This suggest there is a developing trend for married couples to delay their first birth, despite the proportion of women using modern contraceptives not changing from 2006 to 2016 (Diamond‐Smith et al., 2020; Ministry of Health and Population (MOHP), 2007, 2017). Furthermore, no significant association was observed directly between age at pregnancy and preterm delivery. Future research should include investigation of both age at marriage and pregnancy, particularly in regions with high rates of both, to determine where to focus efforts to improve the health and wellbeing of mothers and their babies.

Our finding that there is no increased risk of preterm delivery among those entering into pregnancy at a very young age contrasts with previous research (Althabe et al., 2015; Ganchimeg et al., 2014; Gurung et al., 2020; Kumar et al., 2018; Stewart et al., 2007). The assumption that adolescent pregnancy is detrimental to a woman's reproductive health may not apply to all birth outcomes. In fact, some research suggests that young adolescents deliver better than older adolescents, with a lower risk of obstetrical intervention (Robillard et al., 2019). While adolescent pregnancy has been associated with reduced linear growth, depletion of fat stores and increased nutritional vulnerability due to the nutritional competition between the mother and fetus, our findings suggest that this vulnerability does not increase the risk of delivering preterm (Rah et al., 2008; Wells et al., 2012; Wells et al., 2021). Furthermore, previous research refers to the social risks of adolescent pregnancy, such as increased isolation and exclusion from healthcare facilities (Ganchimeg et al., 2014; Leftwich & Alves, 2017). However, early childbearing in South Asia is generally considered a successful outcome, with extended family members supporting both mother and baby, negating some of the social and environmental problems associated with adolescent pregnancy in other countries (Choe et al., 2005). It is also possible that publication bias exists within the existing literature on associations between adolescent pregnancy and preterm delivery, in which negative findings are withheld from publication (Joober et al., 2012).

While age at first pregnancy did not have a significant association with preterm delivery, gynecological age, which represents the time since menarche rather than chronological age, may still play a role. In the two‐to‐three years following menarche, nutritional competition diminishes, cervix length increases, vaginal pH becomes more acidic, and menstrual frequency increases, all of which contribute to reproductive maturity and are associated with a reduced risk of preterm delivery (Brabin et al., 2005; D'Agostini et al., 2013; Stevens‐Simon et al., 2002). As menarche has been reported to start later in girls with a poor nutritional status, girls may only begin menstruation during their late teens and still be gynecologically immature at age 18 (in the reference category in our models; Gibbs et al., 2012; Stevens‐Simon et al., 2002). Girls experiencing menarche during later adolescence may also contribute to the disconnect between age at marriage and pregnancy. We were not able to assess the association between gynecological maturity and preterm delivery as the LBWSAT did not collect data on age at menarche. As there is a considerable gap in the data on girls' age at menarche and how it is associated with pregnancy outcomes, improved data collection on age at menarche would better inform our understanding of this topic and help determine when women are physiologically ready for pregnancy (Leone & Brown, 2020).

### 5.6 Strengths and limitations

There are several strengths of this study. The high proportion (90%) of participants marrying under the age of 18 years in this study has enabled us to shed new light on this issue; we were able to observe associations with preterm delivery according to the specific age at which girls were married during childhood, whereas previous studies have categorized participants as marrying either under or over the age of 18 years (Huang et al., 2021; Pandya & Bhanderi, 2015; Rahman et al., 2018). Also, our large sample size enabled a thorough analysis of the consequences of marrying and falling pregnant at specific ages during adolescence. The richness of the data collected also enabled successful adjustment for confounders. More broadly, by disentangling the consequences of early marriage and early first pregnancy, we have shed new light on the interpretation of existing studies in this field and highlighted the importance of considering the social and physiological and determinants of health.

The inferences from our research are limited by the constraints of the study. Firstly, gestation length was determined from maternal recall of LMP, which is prone to error. To account for this, we conducted analysis based on the binary outcome of deliveries being term or preterm, but in doing so, we lost information with regard to the extent of prematurity (extremely‐, very‐ or late‐preterm deliveries, or even early term). Furthermore, a substantial proportion of the study population had implausible GL data and were therefore excluded from analysis. Second, due to unforeseen difficulties experienced by the research team during data collection, there was a relatively high proportion of loss to follow‐up resulting in missing gestational age data. Significant differences between those with missing or excluded GL data may have introduced bias into our findings. However, results including and excluding multivariate imputation methods to account for missing data found similar results. Third, the lack of an indicator for psychological stress limits the conclusions of this work, and future work should include such variables in models. Despite these limitations, our data are well suited to provide new insights into the consequences of child marriage and the mechanisms leading to preterm delivery.

## 6 CONCLUSION

Our study found a significant association between marrying during early adolescence, at age ≤14 years, and the likelihood of preterm delivery. Intriguingly, we found no such association between age at first pregnancy and preterm delivery, suggesting the association between child marriage and preterm delivery is driven by factors independent of those associated with early childbearing. Preterm delivery remains the leading cause of death for children before the age of 5 years, therefore efforts to prevent child marriage may result in reduced child mortality. As child marriage is a time of significant emotional distress, psychological stress may be a more important factor than reproductive physiology in the link between age at marriage and preterm delivery. More research into the mental health and nutritional consequences of child marriage will inform our understanding of its consequences and the mechanisms resulting in preterm delivery. Our study is unique due to the high proportion of child brides and the normalization of child marriage in lowland Nepal. Further research to determine the preterm delivery risk associated with early marriage within other populations, where different profiles of child marriage are observed, would elucidate our findings, and improve their generalisability.

## AUTHOR CONTRIBUTIONS

**Akanksha A. Marphatia:** Conceptualization (supporting). **Jonathan C. Wells:** Conceptualization (supporting). **Mario Cortina‐Borja:** Conceptualization (supporting); methodology (supporting). **Dharma S. Manandhar:** Investigation (supporting). **Naomi Saville:** Conceptualization (equal); data curation (supporting); formal analysis (supporting); investigation (lead); methodology (supporting); supervision (supporting); validation (supporting); visualization (supporting).

## CONFLICT OF INTEREST

The authors declare no competing interests.

## Supporting information


**Supplemental Figure1** Directed acyclic graph for the association between age at marriage and preterm delivery for primigravida participants.
**Supplemental Figure 2.** Directed acyclic graph for the association between age at marriage and preterm delivery for multigravida participants.
**Supplemental Figure 3.** Directed acyclic graph for the association between age at first pregnancy and preterm delivery for primigravida participants.
**Supplemental Figure 4.** Directed acyclic graph for the association between age at first pregnancy and preterm delivery for multigravida participants.

**Supplemental Table 1** Characteristics of all participants and those with missing and excluded gestation length data.

**Supplemental Table 2** Associations between age at marriage and age at first pregnancy and preterm delivery, without the use of multiple imputation to account for missing data.

**Supplemental Table 3** Associations between age at marriage and preterm delivery, including height or BMI in models.

**Supplemental Table 4** Associations between age at marriage and preterm delivery, for the subsample for whom height and BMI/MUAC data is available.

**Supplemental Table 5** Associations between age at marriage and age at first pregnancy and preterm delivery, excluding those married >5 years ago.

## Data Availability

The data that support the findings of this study are available on request from the corresponding author. The data are not publicly available due to privacy or ethical restrictions.
