# Supplementary material for: Associations between early marriage and preterm delivery: Evidence from lowland Nepal
Source: Am J Hum Biol. 2021 Dec 4;34(5):e23709. doi: 10.1002/ajhb.23709 (PMC11475576; doi:10.1002/ajhb.23709)
Supplement: Supplementary file 1 — Supplemental Figure1 Directed acyclic graph for the association between age at marriage and preterm delivery for primigravida participants. Supplemental Figure 2. Directed acyclic graph for the association between age at marriage and preterm delivery for multigravida participants. Supplemental Figure 3. Directed acyclic graph for the association between age at first pregnancy and preterm delivery for primigravida participants. Supplemental Figure 4. Directed acyclic graph for the association between age at first pregnancy and preterm delivery for multigravida participants. [file AJHB-34-e23709-s004.docx]

**Supplemental Figure 1.** Directed acyclic graph for the association between age at marriage and preterm delivery for primigravida participants.


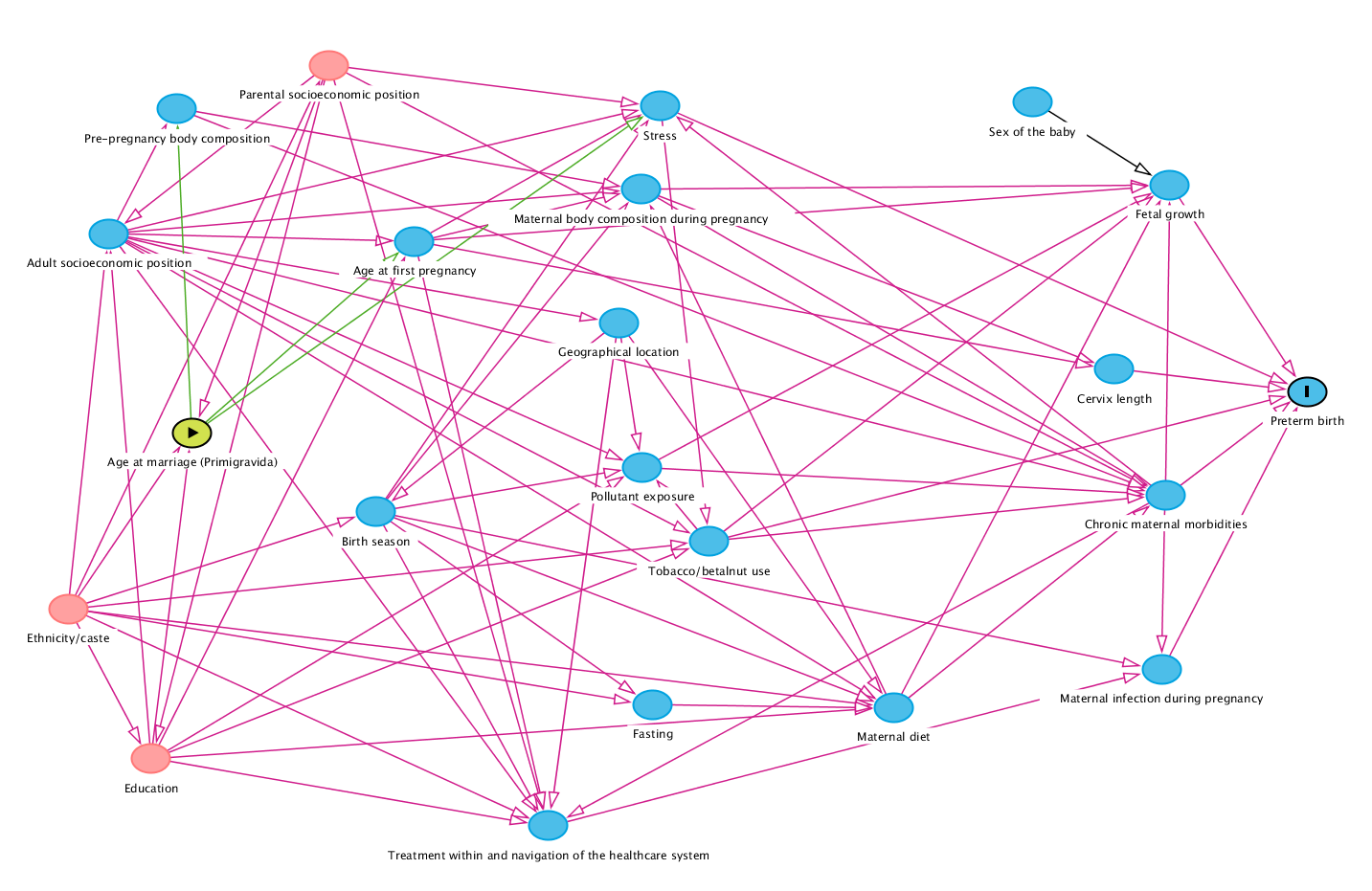


Directed acyclic graph for the association between age at marriage and preterm delivery for primigravida participants.

The arrows in the DAG are entered to represent the hypothesised direct causal effect of one variable on another. Variables that were hypothesised to be directly antecedent to the exposure (age at marriage) and outcome (preterm birth) variables are indicated in pink and identified as a potential confounder. This identified socioeconomic status, ethnicity/caste and education as potential confounders.

*Green node with triangle: exposure, blue node with I: outcome, blue node: ancestor of outcome, pink node: ancestor of exposure and outcome (confounder), green connecting line: causal path, pink connecting line: biasing path.*

**Supplemental Figure 2.** Directed acyclic graph for the association between age at marriage and preterm delivery for multigravida participants.


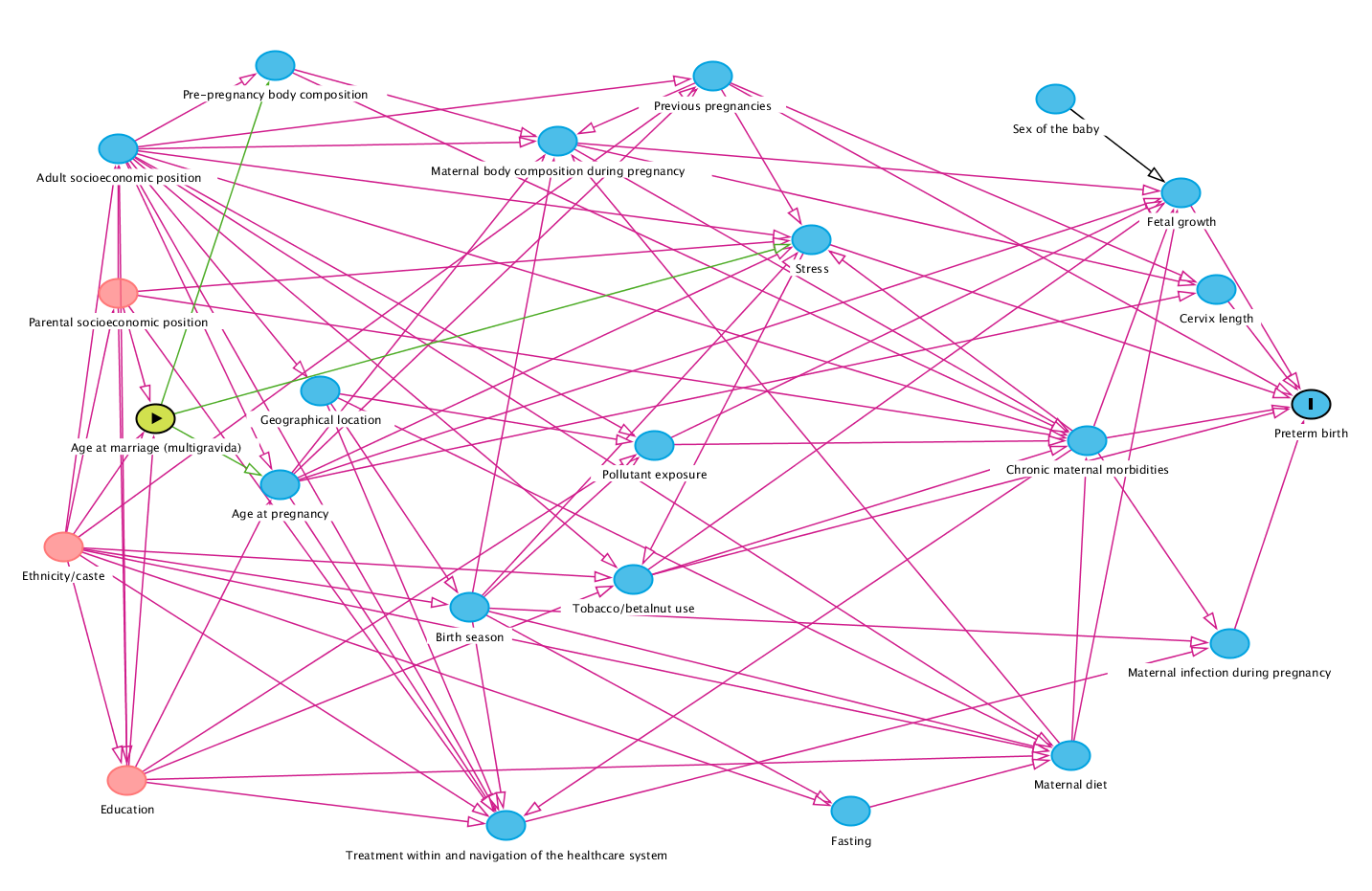


Directed acyclic graph for the association between age at marriage and preterm delivery for multigravida participants. Variables that were hypothesised to be directly antecedent to the exposure (age at marriage) and outcome (preterm birth) variables are indicated in pink and identified as a potential confounder. This identified socioeconomic status, ethnicity/caste and education as potential confounders.

*Green node with triangle: exposure, blue node with I: outcome, blue node: ancestor of outcome, pink node: ancestor of exposure and outcome (confounder), green connecting line: causal path, pink connecting line: biasing path.*

**Supplemental Figure 3.** Directed acyclic graph for the association between age at first pregnancy and preterm delivery for primigravida participants.


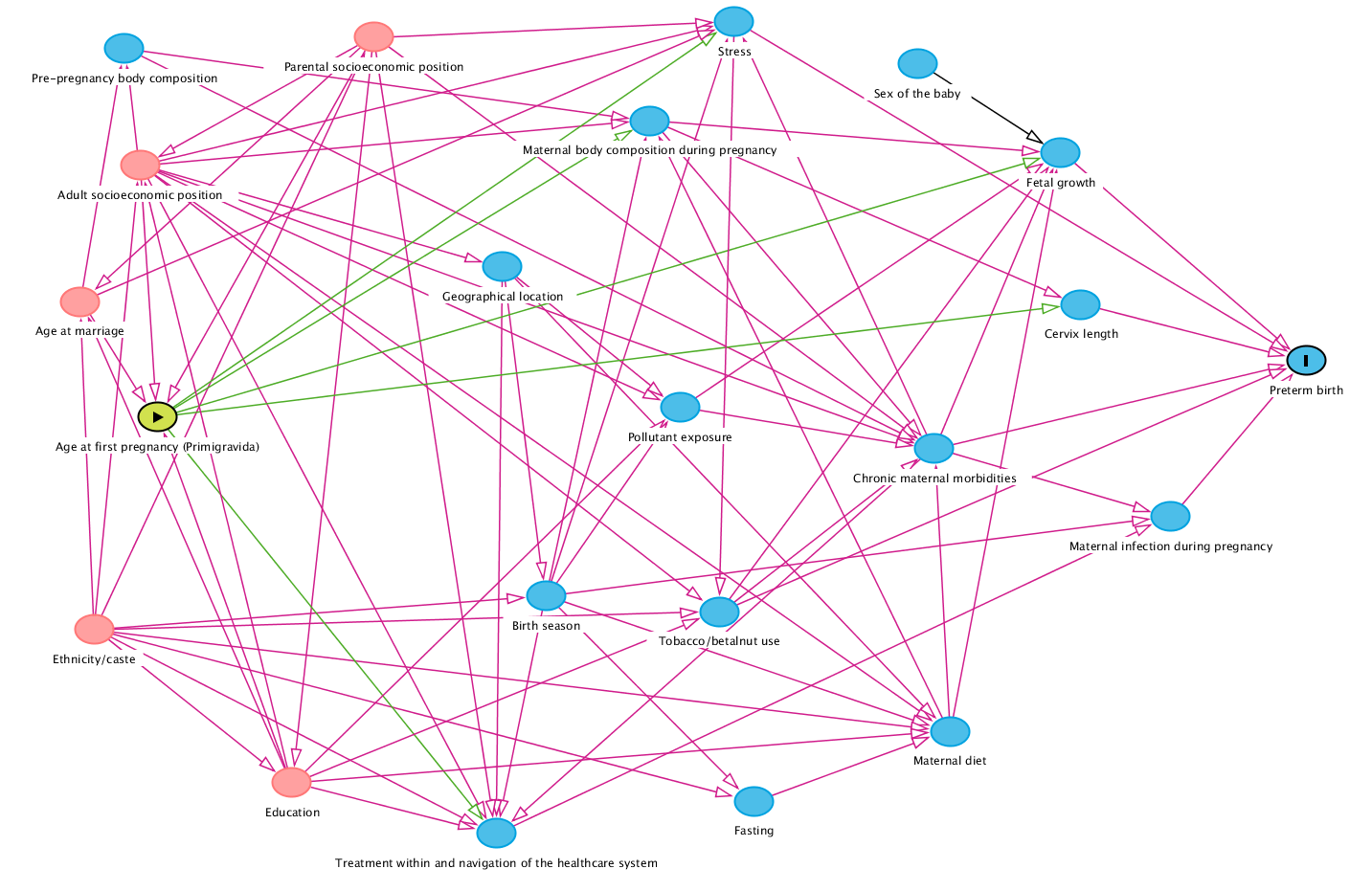


Directed acyclic graph for the association between age at first pregnancy and preterm delivery for primigravida participants. Variables that were hypothesised to be directly antecedent to the exposure (age at first pregnancy) and outcome (preterm birth) variables are indicated in pink and identified as a potential confounder. This identified socioeconomic status, age at marriage, ethnicity/caste and education as potential confounders.

*Green node with triangle: exposure, blue node with I: outcome, blue node: ancestor of outcome, pink node: ancestor of exposure and outcome (confounder), green connecting line: causal path, pink connecting line: biasing path.*

**Supplemental Figure 4.** Directed acyclic graph for the association between age at first pregnancy and preterm delivery for multigravida participants.


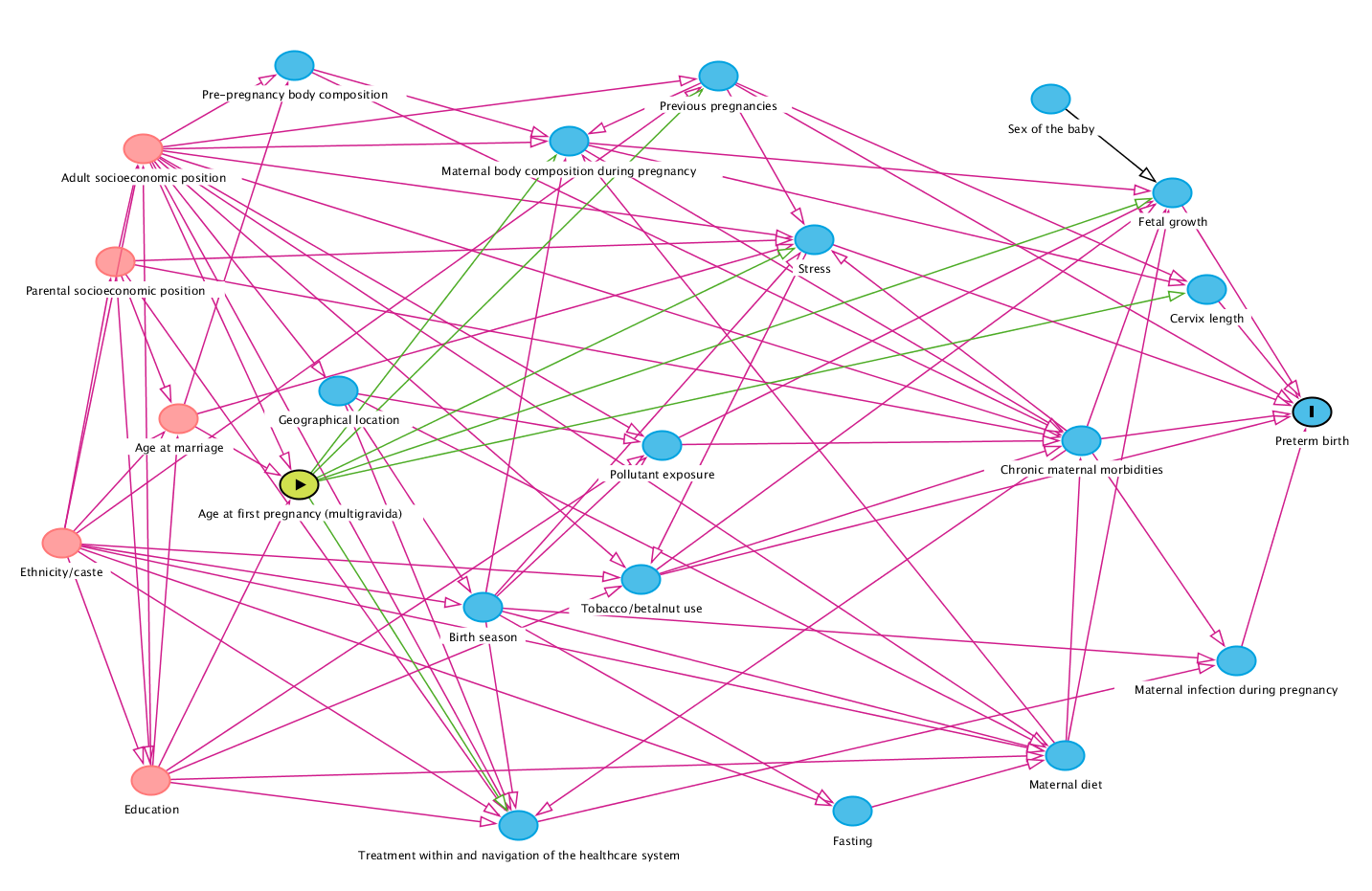


Directed acyclic graph for the association between age at first pregnancy and preterm delivery for multigravida participants. Variables that were hypothesised to be directly antecedent to the exposure (age at first pregnancy) and outcome (preterm birth) variables are indicated in pink and identified as a potential confounder. This identified socioeconomic status, age at marriage, ethnicity/caste and education as potential confounders.

*Green node with triangle: exposure, blue node with I: outcome, blue node: ancestor of outcome, pink node: ancestor of exposure and outcome (confounder), green connecting line: causal path, pink connecting line: biasing path.*
