# Supplementary material for: Associations between early marriage and preterm delivery: Evidence from lowland Nepal
Source: Am J Hum Biol. 2021 Dec 4;34(5):e23709. doi: 10.1002/ajhb.23709 (PMC11475576; doi:10.1002/ajhb.23709)
Supplement: Supplementary file 2 — Supplemental Table 1 Characteristics of all participants and those with missing and excluded gestation length data. [file AJHB-34-e23709-s006.docx]

**Supplemental Table 1**. Characteristics of all participants and those with missing and excluded gestation length data.

|  | Included in analysis | Gestation length missing | | Gestation length excluded | |
| --- | --- | --- | --- | --- | --- |
|  | *n* = 17,974 | *n* = 5,394 | *p-*value | *n* = 1,722 | *p-*value |
| Maternal age at first pregnancy |  |  | 0.002 |  | 0.11 |
| 10-15 y | 19.5% | 17.2% |  | 21.2% |  |
| 16-17 y | 38.8% | 39.3% |  | 36.4% |  |
| 18 y and over | 41.7% | 43.5% |  | 42.4% |  |
| Missing | *2.0%* | *18.7%* |  | *6.0%* |  |
| Maternal age at marriage |  |  | <0.001 |  | 0.71 |
| ≤14 y | 33.8% | 28.4% |  | 34.8% |  |
| 15 y | 26.0% | 25.9% |  | 25.9% |  |
| 16-17 y | 29.9% | 32.4% |  | 28.6% |  |
| 18 y and over | 10.3% | 13.3% |  | 10.8% |  |
| Missing | *18.4%* | *20.7%* |  | *21.9%* |  |
| Caste 3 groups |  |  | <0.001 |  | <0.001 |
| Dalit/Muslim – Disadvantaged | 34.8% | 36.2% |  | 43.5% |  |
| Janjati/Other Terai castes - Middle | 43.1% | 39.4% |  | 37.7% |  |
| Yadav/Brahmin - Advantaged | 22.1% | 24.5% |  | 18.8% |  |
| Missing | *0.0%* | *0.7%* |  | *0.0%* |  |
| Household asset score |  |  | <0.001 |  | <0.001 |
| 1 - Most deprived | 19.9% | 18.3% |  | 25.3% |  |
| 2 | 20.3% | 18.7% |  | 20.3% |  |
| 3 | 20.3% | 20.0% |  | 19.2% |  |
| 4 | 20.0% | 21.0% |  | 19.3% |  |
| 5 - Least deprived | 19.5% | 22.0% |  | 15.8% |  |
| Missing | *1.2%* | *17.5%* |  | *4.2%* |  |
| Mother education level |  |  | <0.001 |  | <0.001 |
| Never went to school | 64.7% | 58.4% |  | 73.3% |  |
| Primary | 10.3% | 10.9% |  | 9.3% |  |
| Lower secondary | 9.9% | 11.4% |  | 7.5% |  |
| Secondary and above | 15.1% | 19.3% |  | 9.9% |  |
| Missing | *0.1%* | *16.0%* |  | *3.0%* |  |
| Study arm woman enrolled in |  |  | <0.001 |  | 0.009 |
| Control | 22.5% | 17.0% |  | 20.2% |  |
| Women's group | 23.6% | 18.7% |  | 21.7% |  |
| Cash | 27.6% | 33.1% |  | 30.1% |  |
| Food | 26.3% | 31.2% |  | 28.0% |  |
| Missing | *0.0%* | *0.0%* |  | *0.0%* |  |
| Season of birth |  |  | - |  | <0.001 |
| Winter: Mid-December to mid-March | 29.1% | - |  | 23.6% |  |
| Spring: Mid-March to mid-June | 21.8% | - |  | 23.9% |  |
| Monsoon: Mid-June to mid-September | 26.7% | - |  | 29.0% |  |
| Autumn: Mid-September to mid-November | 22.3% | - |  | 23.5% |  |
| Missing | *2.1%* | *100.0%* |  | *3.0%* |  |
| Sex of infant |  |  | 0.93 |  | 0.012 |
| Boy | 53.1% | 50.0% |  | 49.9% |  |
| Girl | 46.9% | 50.0% |  | 50.1% |  |
| Missing | *1.0%* | *100.0%* |  | *1.0%* |  |

Legend: Displays characteristics of all participants included in the analysis, alongside the characteristics of participants with missing and excluded gestation length (GL) data. For categorical variables, column percentages are displayed, and *p-*values are displayed from chi-squared tests comparing those with missing or excluded GL data with those included in the analysis. % missing data for each variable is reported. Percentages for the seasonality of birth for participants missing GL data have been omitted as they could not be calculated due to the lack of date of birth.

*n, sample size; y, years of age.*
