# Supplementary material for: Associations between early marriage and preterm delivery: Evidence from lowland Nepal
Source: Am J Hum Biol. 2021 Dec 4;34(5):e23709. doi: 10.1002/ajhb.23709 (PMC11475576; doi:10.1002/ajhb.23709)
Supplement: Supplementary file 3 — Supplemental Table 2 Associations between age at marriage and age at first pregnancy and preterm delivery, without the use of multiple imputation to account for missing data. [file AJHB-34-e23709-s001.docx]

**Supplemental table 2.** Associations between age at marriage and age at first pregnancy and preterm delivery, without the use of multiple imputation to account for missing data

| Age at marriage | | | | | | | |
| --- | --- | --- | --- | --- | --- | --- | --- |
| Multigravida | | | | Primigravida | | | |
| 1. Association between age at marriage and preterm delivery. aOR; adjusted for confounders | | | | | | | |
|  | aOR (95% CI) | p-value |  | | aOR (95% CI) | *p*-value |  |
| Age at marriage  *n* = 10,085 |  |  | Age at marriage  *n* = 5,447 | |  |  |  |
| ≤14 y | 0.93 (0.76 -1.14) | *0.484* | ≤14 y | | 1.31 (1.02 -1.67) | *0.032** |  |
| 15 y | 0.98 (0.80 -1.21) | *0.879* | 15 y | | 1.13 (0.89 -1.45) | *0.306* |  |
| 16-17 y | 1.02 (0.83 -1.25) | *0.873* | 16-17 y | | 1.14 (0.91 -1.43) | *0.240* |  |
| ≥ 18 y | 1.0 (ref) |  | ≥ 18 y | | 1.0 (ref) |  |  |
| 1. Association between age at marriage and preterm delivery. aOR; adjusted for confounders, plus age at current pregnancy to assess for mediation | | | | | | | |
|  | aOR (95% CI) | p-value |  | | aOR (95% CI) | *p*-value |  |
| Age at marriage  *n* = 10,019 |  |  | Age at marriage  *n* = 5,447 | |  |  |  |
| ≤14 y | 0.88 (0.69 -1.1) | *0.263* | ≤14 y | | 1.31 (1.01 -1.71) | *0.045** |  |
| 15 y | 0.93 (0.74 -1.17) | *0.548* | 15 y | | 1.18 (0.91 -1.53) | *0.214* |  |
| 16-17 y | 0.99 (0.8 -1.23) | *0.921* | 16-17 y | | 1.19 (0.94 -1.50) | *0.146* |  |
| ≥ 18 y | 1.0 (ref) |  | ≥ 18 y | | 1.0 (ref) |  |  |
| Age at first pregnancy | | | | | | | |
| Multigravida | | | | Primigravida | | | |
| Association between age at first pregnancy and preterm delivery. aOR; adjusted for confounders | | | | | | | |
|  | aOR (95% CI) | p-value |  | | aOR (95% CI) | *p*-value |  |
| Age at first pregnancy n = 10,019 |  |  | Age at first pregnancy  *n* = 5,447 | |  |  |  |
| 10-15 y | 1.09 (0.93 -1.29) | *0.289* | 10-15 y | | 1.08 (0.83 -1.42) | *0.566* |  |
| 16-17 y | 1.09 (0.96 -1.25) | *0.178* | 16-17 y | | 0.92 (0.78 -1.07) | *0.285* |  |
| ≥ 18 y | 1.0 (ref) |  | ≥ 18 y | | 1.0 (ref) |  |  |
|  |  |  |  | |  |  |  |

Legend: Association between preterm delivery and age at marriage and age at first pregnancy for multigravida and primigravida women.
Core confounders identified using a directed acyclic graph were maternal caste, maternal education and household asset score, plus age at marriage for the association between age at first pregnancy and preterm delivery.

Age at marriage models adjusted for:

1. Cluster, study arm, strata, and core confounders
2. Cluster, study arm, strata, and core confounders + age at current pregnancy

Age at pregnancy model adjusted for cluster, study arm, strata, and core confounders.

*n, sample size; aOR, adjusted odds ratio; 95% CI, 95% confidence interval; y, years of age.
p-value significance: * <0.05, ***<0.01*
