# Supplementary material for: Associations between early marriage and preterm delivery: Evidence from lowland Nepal
Source: Am J Hum Biol. 2021 Dec 4;34(5):e23709. doi: 10.1002/ajhb.23709 (PMC11475576; doi:10.1002/ajhb.23709)
Supplement: Supplementary file 4 — Supplemental Table 3 Associations between age at marriage and preterm delivery, including height or BMI in models. [file AJHB-34-e23709-s002.docx]

**Supplemental table 3** Associations between age at marriage and preterm delivery, including height or BMI in models.

| Age at marriage | | | | | | | |
| --- | --- | --- | --- | --- | --- | --- | --- |
| Multigravida | | | | Primigravida | | | |
| Association between age at marriage and preterm delivery, including height. aOR; adjusted for confounders | | | | | | | |
|  | aOR (95% CI) | p-value |  | | aOR (95% CI) | | *p*-value |
| Age at marriage  *n* = 7,931 |  |  | Age at marriage  *n* = 3,795 | |  | |  |
| ≤14 y | 0.93 (0.74 -1.16) | *0.509* | ≤14 y | | 1.36 (1.01 -1.82) | | *0.042** |
| 15 y | 0.93 (0.73 -1.18) | *0.558* | 15 y | | 1.05 (0.78 -1.41) | | *0.755* |
| 16-17 y | 0.95 (0.74 -1.21) | *0.667* | 16-17 y | | 1.01 (0.77 -1.33) | | *0.929* |
| ≥ 18 y | 1.0 (ref) |  | ≥ 18 y | | 1.0 (ref) | |  |
| Association between age at marriage and preterm delivery, including BMI. aOR; adjusted for confounders. | | | | | | | |
|  | aOR (95% CI) | p-value |  | | aOR (95% CI) | | *p*-value |
| Age at marriage  *n* = 1,960 |  |  | Age at marriage  *n* = 1,074 | |  | |  |
| ≤14 y | 1.09 (0.69 -1.73) | *0.714* | ≤14 y | | 2.15 (1.13 -4.09) | | *0.020** |
| 15 y | 0.90 (0.55 -1.47) | *0.676* | 15 y | | 1.70 (0.90 -3.20) | | *0.101* |
| 16-17 y | 0.96 (0.59 -1.56) | *0.867* | 16-17 y | | 1.78 (0.99 -3.17) | | *0.052* |
| ≥ 18 y | 1.0 (ref) |  | ≥ 18 y | | 1.0 (ref) | |  |
| Association between age at marriage and preterm delivery, including MUAC. aOR; adjusted for confounders. | | | | | | | |
|  | aOR (95% CI) | p-value |  | | aOR (95% CI) |  | |
| Age at marriage  *n* = 1,960 |  |  | Age at marriage  *n* = 1,074 | |  |  | |
| ≤14 y | 1.10 (0.69 -1.75) | *0.685* | ≤14 y | | 2.06 (1.08 -3.94) | *0.028** | |
| 15 y | 0.92 (0.56 -1.50) | *0.731* | 15 y | | 1.67 (0.89 -3.15) | *0.111* | |
| 16-17 y | 0.96 (0.59 -1.56) | *0.866* | 16-17 y | | 1.73 (0.97 -3.09) | *0.064* | |
| ≥ 18 y | 1.10 (0.69 -1.75) |  | ≥ 18 y | | 2.06 (1.08 -3.94) |  | |

Legend: Association between preterm delivery and age at marriage for multigravida and primigravida women, adjusting for height and body mass index to assess for the role of nutritional markers.

Models were adjusted for cluster, study arm, strata, core confounders, and either height (collected at any time during pregnancy), maternal BMI (collected during early pregnancy) or maternal mid upper-arm circumference (MUAC; collected during early pregnancy).

Core confounders identified using a directed acyclic graph were maternal caste, maternal education, and household asset score, plus age at marriage for the association between age at first pregnancy and preterm delivery.

*BMI, body mass index; MUAC, mid upper arm circumference; n, sample size; aOR, adjusted odds ratio; 95% CI, 95% confidence interval; y, years of age.
p-value significance: * <0.05, ***<0.01*
