# Supplementary material for: Associations between early marriage and preterm delivery: Evidence from lowland Nepal
Source: Am J Hum Biol. 2021 Dec 4;34(5):e23709. doi: 10.1002/ajhb.23709 (PMC11475576; doi:10.1002/ajhb.23709)
Supplement: Supplementary file 5 — Supplemental Table 4 Associations between age at marriage and preterm delivery, for the subsample for whom height and BMI/MUAC data is available. [file AJHB-34-e23709-s005.docx]

**Supplemental table 4** Associations between age at marriage and preterm delivery, for the subsample for whom height and BMI/MUAC data is available

| Age at marriage | | | | | | |
| --- | --- | --- | --- | --- | --- | --- |
| Multigravida | | | | Primigravida | | |
| Association between age at marriage and preterm delivery, for the sub-sample with data on height. aOR; adjusted for confounders | | | | | | |
|  | aOR (95% CI) | p-value |  | | aOR (95% CI) | *p*-value |
| Age at marriage  *n* = 7,931 |  |  | Age at marriage  *n* = 3,795 | |  |  |
| ≤14 y | 0.93 (0.74 -1.17) | *0.519* | ≤14 y | | 1.36 (1.01 -1.83) | *0.040** |
| 15 y | 0.93 (0.73 -1.18) | *0.566* | 15 y | | 1.05 (0.78 -1.41) | *0.735* |
| 16-17 y | 0.95 (0.74 -1.21) | *0.666* | 16-17 y | | 1.01 (0.77 -1.33) | *0.920* |
| ≥ 18 y | 1.0 (ref) |  | ≥ 18 y | | 1.0 (ref) |  |
| Association between age at marriage and preterm delivery, for the sub-sample with data on BMI and MUAC. aOR; adjusted for confounders. | | | | | | |
|  | aOR (95% CI) | p-value |  | | aOR (95% CI) | *p*-value |
| Age at marriage  *n* = 1,960 |  |  | Age at marriage  *n* = 1,074 | |  |  |
| ≤14 y | 1.09 (0.69 -1.73) | *0.714* | ≤14 y | | 2.09 (1.10 -3.97) | *0.025** |
| 15 y | 0.90 (0.55 -1.47) | *0.678* | 15 y | | 1.68 (0.89 -3.17) | *0.107* |
| 16-17 y | 0.96 (0.59 -1.56) | *0.860* | 16-17 y | | 1.74 (0.98 -3.11) | *0.061* |
| ≥ 18 y | 1.0 (ref) |  | ≥ 18 y | | 1.0 (ref) |  |

Legend: Association between preterm delivery and age at marriage for multigravida and primigravida women for the sub-sample with height or BMI/MUAC available, for comparison with the models adjusting for nutritional status (NB. not including adjustment for nutritional status).

Models were adjusted for cluster, study arm, strata, and core confounders. Core confounders identified using a directed acyclic graph were maternal caste, maternal education, and household asset score, plus age at marriage for the association between age at first pregnancy and preterm delivery.

*BMI, body mass index; MUAC, mid upper arm circumference; n, sample size; aOR, adjusted odds ratio; 95% CI, 95% confidence interval; y, years of age.
p-value significance: * <0.05, ***<0.01*

Inclusion and exclusion of nutritional markers in models on the sub-sample of participants with nutritional markers available exerts little change on the association between age at marriage and preterm delivery. This suggests that the nutritional status of the mother is not driving this association. However, these are all markers of nutritional status before pregnancy and we can therefore not rule-out the any changes during pregnancy.
